# Doorways do not always cause forgetting: a multimodal investigation

**DOI:** 10.1186/s40359-021-00536-3

**Published:** 2021-03-08

**Authors:** Jessica McFadyen, Christopher Nolan, Ellen Pinocy, David Buteri, Oliver Baumann

**Affiliations:** 1 Queensland Brain Institute, University of Queensland, Brisbane, QLD 4072 Australia; 2 Max Planck UCL Centre for Computational Psychiatry and Ageing Research, University College London, London, UK; 3 School of Psychology and Interdisciplinary Centre for the Artificial Mind, Bond University, Robina, QLD 4226 Australia

**Keywords:** Doorway effect, Memory, Spatial navigation, Event boundary

## Abstract

**Background:**

The ‘doorway effect’, or ‘location updating effect’, claims that we tend to forget items of recent significance immediately after crossing a boundary. Previous research suggests that such a forgetting effect occurs both at physical boundaries (e.g., moving from one room to another via a door) and metaphysical boundaries (e.g., imagining traversing a doorway, or even when moving from one desktop window to another on a computer). Here, we aimed to conceptually replicate this effect using virtual and physical environments.

**Methods:**

Across four experiments, we measured participants’ hit and false alarm rates to memory probes for items recently encountered either in the same or previous room. Experiments 1 and 2 used highly immersive virtual reality without and with working memory load (Experiments 1 and 2, respectively). Experiment 3 used passive video watching and Experiment 4 used active real-life movement. Data analysis was conducted using frequentist as well as Bayesian inference statistics.

**Results:**

Across this series of experiments, we observed no significant effect of doorways on forgetting. In Experiment 2, however, signal detection was impaired when participants responded to probes after moving through doorways, such that false alarm rates were increased for mismatched recognition probes. Thus, under working memory load, memory was more susceptible to interference after moving through doorways.

**Conclusions:**

This study presents evidence that is inconsistent with the location updating effect as it has previously been reported. Our findings call into question the generalisability and robustness of this effect to slight paradigm alterations and, indeed, what factors contributed to the effect observed in previous studies.

## Background

Our experience of the world is continuous and rich with information. To manage this constant stream of information, we segment our experience into events, which are stored as episodic memory for later retrieval [22]. Events are determined by boundaries, denoting the beginning and end of a particular period of time. Salient environmental changes are thought to dictate the location of event boundaries (e.g., a change in location, a shift in goal, etc.; [23].

A commonly encountered event boundary is a doorway. Previous research has demonstrated that long-term memory for the temporal order of items is better for items presented within the same room [4] or context [2, 22] than for items presented across different rooms or contexts. Short-term memory is also reduced for items that were presented before an event boundary. For example, while reading, memory for words preceding the phrase “An hour later” is worse than preceding the phrase “A while later”, as the former is more like an event boundary [21]. Similarly, research suggests that walking through doorways—in reality, virtual reality, and even in our imagination—causes us to forget information obtained in the previous room.

The effect of declined memory performance after passing through a doorway or after another event boundary has come to be known as the location updating effect [16], but is also referred to as the doorway effect or the event horizon effect [14]. In the initial demonstration of the doorway effect [15], participants played a computer game in which they freely navigated a 3D environment. The environment consisted of a series of rooms, each containing a table with an object on top. Participants were tasked with moving each object from one table to the next, which would either be in the next room connected by a door (“shift” condition) or the same room (“no shift” condition). Halfway along the trajectory, participants’ recognition memory was probed with an object description (a colour-shape pair; e.g., “red cube”) that either matched the object they were carrying, the object they had set down on the previous table, or neither. The results of the study revealed that, after passing through a doorway, participants would more often fail to recognise the probes (reflected by a reduced hit rate) for the objects they were carrying than if they had not passed through a doorway [15].

Numerous iterations of this experiment have explored the robustness of the doorway effect. These studies have found that the effect persists regardless of the type of probe (text vs. images [18], recognition vs. recall [10]), travel time [8, 9], the level of immersion (small screens, big screens, or real-life environments [16]; active vs. passive interaction; [11], real or imagined; [6, 12]), age [17], whether the dividing wall is transparent or opaque [8], whether there were additional items to remember [18], or whether participants were probed after returning to the room the item was first encoded in [16].

The underlying cause of the location updating effect is thought to relate to temporal prediction, such that the contents of working memory acquired while in one event is highly predictive while still in that event and lowly predictive of any upcoming new event, which will have its own new set of statistical regularities [19]. Hence, the information is cleared from working memory when the event boundary is crossed [7]. Within this framework, it seems somewhat surprising that the doorway effect is so robust across the literature, as all the events are relatively similar, both in terms of their visual features as well as the participants’ goals (i.e., the only task is to remember the set-down and picked-up objects). Why, then, does the doorway effect persist, even when the predictive validity of task information from previous events is relatively high?

**Fig. 1:**
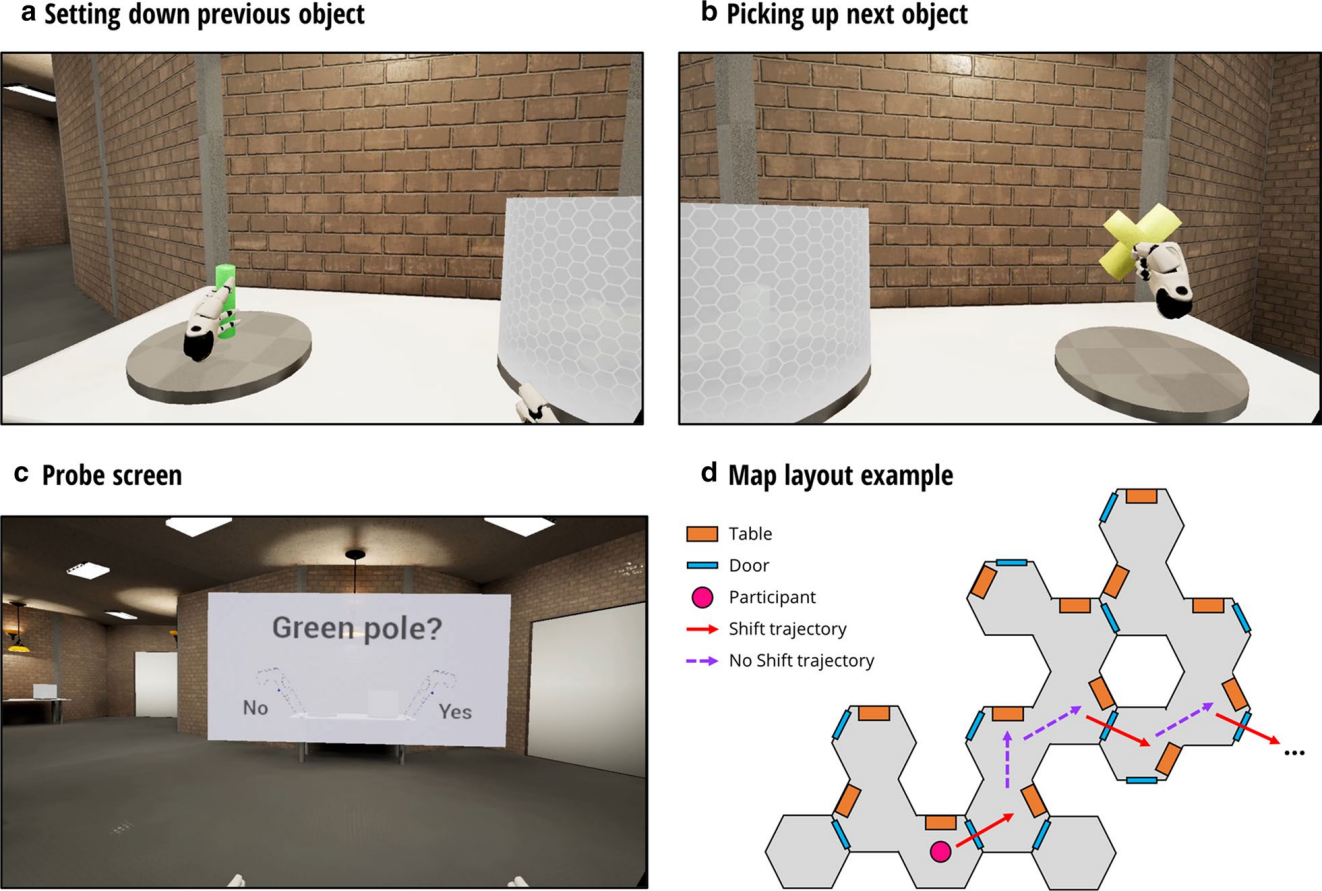
Screenshots and Layout of the Custom-made Virtual Environment for Experiments 1 and 2. (**a**) After being moved to a new table, participants were required to first place the object acquired in the previous room on the new table. This was done by participants reaching behind their head with the left controller and “taking the object out of their backpack” by holding the back trigger, and then releasing the trigger when positioned over the table. (**b**) Participants then picked up the next object and placed it in their backpack, by gripping the back trigger and reaching behind their head, and then releasing the trigger. (**c**) Upon releasing the object into their backpack, participants were passively moved backwards, then turned left or right (either towards a door or towards another part of the room) and moved towards the next table. Halfway along the trajectory, a probe screen appeared with an object description (colour and shape). Participants responded “yes” (right controller) or “no” (left controller) as to whether the probe described either the object that was most recently set down or the object that was most recently picked up. Probes would always be a combination of the colour and shape of the set-down and picked-up object (here, the probes could be: “green pole”, “yellow cross”, “green cross”, or “yellow pole”). (**d**) A bird’s eye view of an example map layout, with 6 trials (“shifts” indicated by solid red arrows and “no shifts” indicated by dashed purple arrows). All images in the figure have been created by the authors

**Fig. 2:**
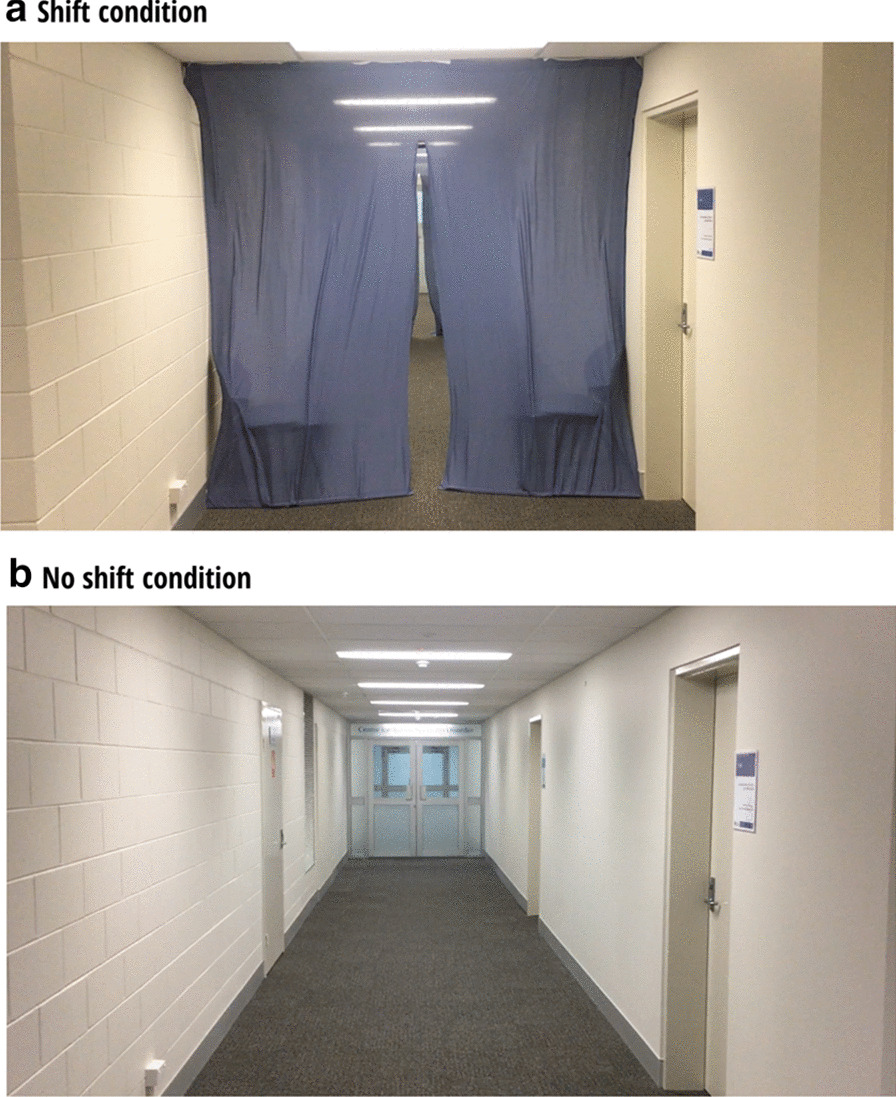
Task set-up and stimuli for Experiments 3 and 4. (**a**) A photograph of the hallway set up for the “shift” condition in both Experiments 3 and 4. Participants would actively walk (Experiment 4) or would passively watch a first-person perspective video of someone walking (Experiment 3) to the end of the hallway and back, while they completed a counting distractor task and simultaneously tried to remember a set of stimuli. (**b**) The same as ***a***, except with the hallway set up for the “no shift” condition. All images in this figure have been created by the authors

The aim of the current study was to examine whether boundaries created by doors induce forgetting under different experimental conditions, ranging from virtual reality to real life movement (see Figs. 1, 2). First, in Experiments 1 and 2, we conceptually replicated key elements of Radvansky and colleagues’ study design while controlling for a number of additional factors (see Experiments 1 and 2: Aim). Second, in Experiments 3 and 4, participants either passively (via video watching) or actively moved through an actual environment with or without a boundary (see Experiments 3 and 4: Aim).

## Experiments 1 and 2

### Aim

The aim of Experiment 1 was to conceptually replicate the original study demonstrating the doorway effect [15] under controlled conditions. We increased immersion by using a full virtual reality headset and designed the virtual environment so that all rooms were visually identical, as opposed to previous studies [15] where the walls were different colours. Thus, in our experiment, any forgetting could only be attributed to boundaries rather than salient changes in context or visual processing. We also more than doubled the number of trials (51 trials to 110) so as to maximise statistical power. We hypothesised that, if the doorway effect is indeed solely attributable to door boundaries rather than extraneous experimental factors, we would observe impaired recognition memory in the form of fewer hits and more false alarms.

In Experiment 2, we incorporated an additional task that increased working memory load, in which participants counted backwards aloud from a given number during the first half of the movement trajectory. The event horizon model stipulates that working memory is updated at boundaries, replacing the previous event model with the new event model [14]. By filling working memory capacity with an extraneous task, we hypothesised that the previous event model would be even more susceptible to being “flushed” from working memory when it is already overloaded [7].

## Methods

### Participants

We estimated the Cohen’s *d* effect size using *d* = M_1_–M_2_/s_pooled_, where s_pooled_ = √[(*s*_1_^2^ + *s*_2_^2^) / 2]). This revealed that the size of the doorway effect across a range of comparable studies was *d* = 0.66 [4, 6, 8–11, 15, 18]. A power analysis revealed that 27 participants would be required for a paired *t*-test with a typical α = 0.05 and a β = 9. Radvansky et al. [16] stated that 16 pairs of participants would be required to detect the doorway effect using an independent-samples t-test. Previous studies report significant effects from samples between 40 and 60 participants [6, 9, 15, 16, 18], as well as smaller samples of 16–30 [10, 11, 17].

For Experiment 1, we recruited 40 participants through the University of Queensland’s paid research participation scheme, which draws from adults within the local community. Participants were compensated AUD$20 per hour for their time and provided written consent. Of the 40 participants, 9 aborted the experiment due to motion sickness and 2 participants were excluded due to poor performance (< 20% accuracy in any condition). This left a final sample of 29 participants, consisting of 13 males and 16 females aged between 18 and 33 years (M = 23, SD = 4.06; age data missing for 1 participant).

For Experiment 2, we recruited 63 first-year psychology students from the University of Queensland who received course credit for their time. All participants provided written consent and were required to have normal or corrected-to-normal vision and normal colour vision. Of the 63 participants, 14 aborted the experiment due to motion sickness, and 4 participants were excluded due to poor performance (< 20% accuracy in any condition). This left a final sample of 45 participants, consisting of 20 males and 25 females aged between 18 and 45 years (M = 23.65, SD = 6.36; age data missing for 11 participants). This study was approved by the University of Queensland’s Human Research Ethics Committee.

### Stimuli and equipment

Two similar virtual environments (one map per block) were created using Unreal Engine 4 (Epic Games, 2019). Participants viewed the environment with an HTC Vive Headset and interacted with the environment using left and right HTC Vive wireless controllers.

Within the virtual environment, participants were situated inside a brick building containing Y-shaped rooms (see Fig. 1). Each room contained a white table, with two grey circular platforms on top. The left platform was empty (for participants to put objects on) and the right platform had an object on top (for participants to pick up). While an object was present on a platform, a white shield became visible to hide the contents from the participant. The shield disappeared upon intersecting with the participant’s virtual hand (controlled by a wireless controller). This was done to equate visual exposure to the set-down and picked-up objects as much as possible.

The 3D objects were created in Blender v2.79 (Blender Institute, Amsterdam). There were 6 different shapes (cube, cone, pole, disc, cross, and wedge) approximately 10 × 10 × 10 cm in size that could be one of 6 colours (red, blue, cyan, green, yellow, and purple), similar to previous studies [15]. The order of objects across trials was pseudorandom so that the same colour or shape could not be repeated and so that there were roughly equal instances of each object shape and colour across a block.

The table was always situated at the top of the Y-shaped room. The two forks of the room always consisted of a wall with a door on one side (“shift” condition) and no wall (“no shift” condition) on the other side. The doors were elevator-style, consisting of two vertical slabs that moved apart upon the participant approaching and passing through. Whether the door was on the left or right was randomly counterbalanced across each map. This was done so that, before picking up the object, participants could not predict whether they would pass through a door or not (and thus there could be no influence of shift on initial memory encoding). This improves upon previous studies [15], where “shift” rooms were small and “no shift” rooms were large with a darkened section, hence a doorway effect could be attributable to either the boundary crossing *or* the way items were initially encoded.

Two different maps were generated, with one used for each block (order counterbalanced across participants). There were 61 rooms in each block, giving 60 transitions (30 shift and 30 no shift).

### Procedure

First, participants were seated at a desk where they provided written consent. The experimenter then verbally explained the task and showed the participants pictures of each object shape and colour (and their corresponding labels) before fitting the HTC VIVE Headset. Participants were virtually moved through the environment while in a seated position. At the beginning of each trial, participants faced a table with an object on top. Participants were instructed to use the right controller to pick up the object (by holding the back trigger button to ‘grip’ the object) and put it in their virtual “backpack” (by moving the controller behind their head and releasing the back trigger). Upon object release, participants were passively moved backwards, turned left or right (either towards a door or towards the other open part of the same room), and then moved towards the next table. Upon reaching the next table, participants took the previous object out of their “backpack” (by reaching behind their head with their left controller and holding the back trigger) and placed it on the empty grey platform on the table (by releasing the back trigger). They were to then repeat the process again by picking up the next object on the right, memorising the object they had just set down (the “dissociated” object) and the one they next pick up (the “associated” object).

Participants’ memory for the associated and dissociated objects was probed by a screen that appeared halfway through the movement trajectory between tables (in a “shift” condition, this occurred immediately after passing through the doors). The screen presented text for a colour (e.g. “blue”) and a shape (e.g. “cube”), followed by a question mark. Underneath were buttons for “yes” and “no” which participants could select with their left or right controller, respectively (no movement required). The colour-shape probes described either the associated object (e.g., “blue cube”), the dissociated object (e.g., “red cone”), or an incorrect combination of the two (e.g., “blue cone” or “red cube”). These latter probes are referred to as “negative” probes. Participants were instructed to answer “yes” if the colour-shape probe matched *either* the associated or dissociated object, and “no” otherwise. Participants were encouraged to maximise accuracy, but also to keep response times short (no feedback was given). Participants were also instructed to keep their eyes open and to not say the object names out loud.

For Experiment 2, a counting task was introduced to increase working memory load. After participants released the object into their inventory, the experimenter provided a random number between 20 and 100 (using a random number generator, with the result spoken aloud). Participants were required to count backwards from the number aloud in steps of 6  (e.g., from 60: “54, 48, 42, 36…”) until the probe screen appeared. Participants were encouraged to count as far back as they could within the time frame (approximately 4 s) while still memorising the two objects as accurately as possible. In block 2, the subtraction value was changed to 7 to prevent repetition. In certain cases, the counting decrement was adjusted after the first block to account for individual differences in mathematical ability. Decrements were made to be easier (to steps of 4 or 5) if participants could only count back to 2 numbers or less (seven participants), or harder (to steps of 13) if participants could count back to 5 numbers or more (six participants). Thus, participants were typically able to count back to 3 or 4 numbers before the probe appeared. The duration of each block was approximately 25 min.

## Results

### Experiment 1

In Experiment 1, we aimed to conduct a highly controlled conceptual replication of the doorway effect by using a highly immersive environment and controlling for elements like context (all rooms were identical) and anticipation (not possible to know doorway condition until after movement initiated). We recorded the accuracy and response time, excluding trials that were longer than 10 s (indicating a pause in the experiment) or shorter than 0.25 s (indicating accidental button press), as well as the first 10 trials of the first block (due to ongoing instruction from the experimenter). We then removed any trials with outlying response times (± 3 SDs from each participant’s mean). This left 100 to 110 trials per participant, with at least 14 trials per condition (M = 17, SD = 1.58).

**Fig. 3:**
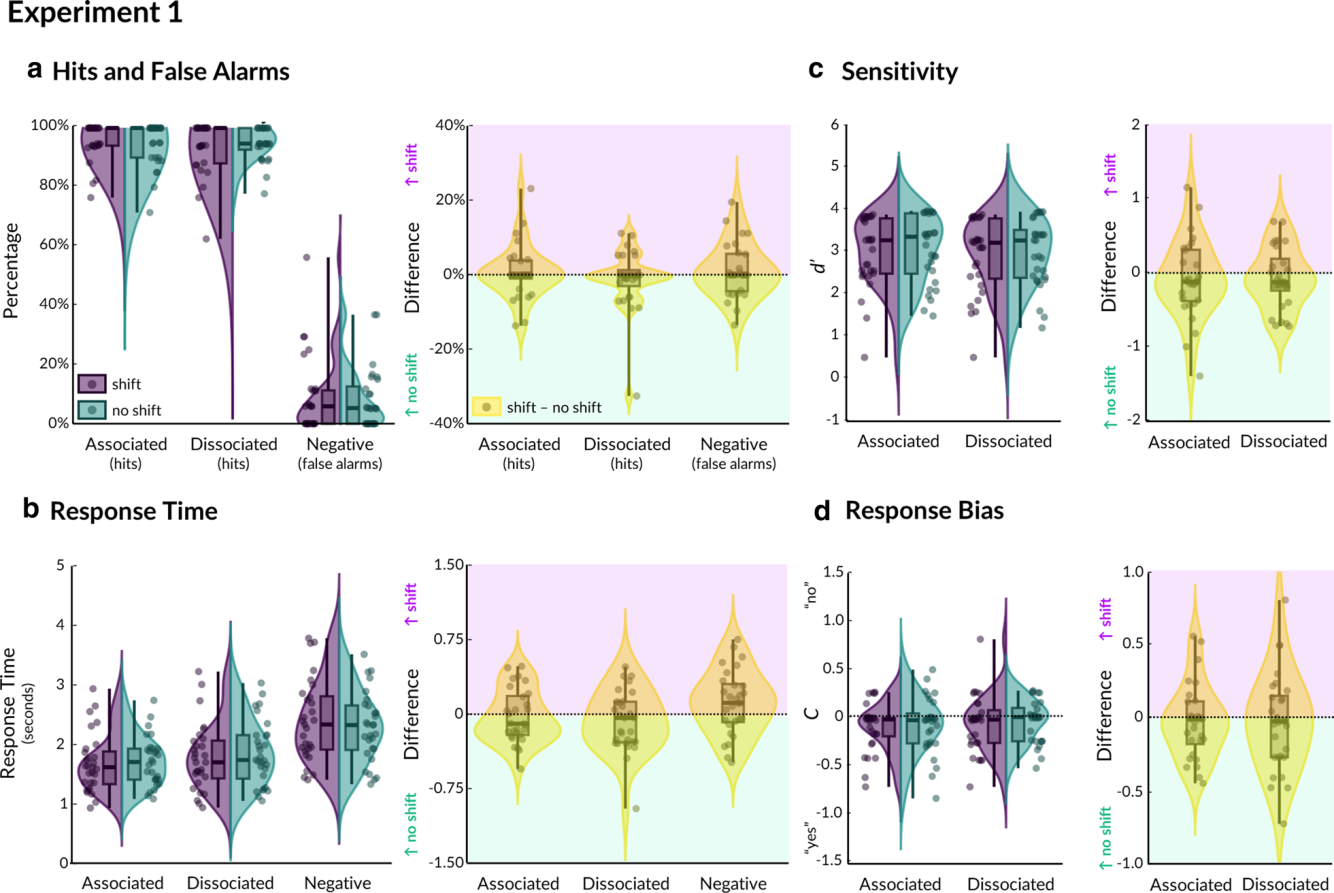
Results from virtual reality Experiment 1. Violin plots are shown for each condition (purple = shift, green = no shift), as well as the difference score per condition (yellow = shift minus no shift). Hits and False alarms are shown in **a**, response times in **b**, signal detection (*d prime*) in **c**, and response bias (*c* parameter) in **d**. Boxplots indicate median, with shaded area for the interquartile range (25th to 75th percentiles), and vertical lines for the minimum and maximum. Dots represent individual participants (*N* = 29)

The mean hit rate across conditions per participant ranged from 81.57% to 100% (M = 94.67%, SD = 5.54%; see Fig. 3a and Table 1). The mean false alarm rate across conditions per participant ranged from 0% to 46.55% (M = 8.31%, SD = 11.05%). We drew upon signal detection theory and calculated the sensitivity index *d’* and the *C* bias parameter of the associated and dissociated probes, per shift condition, using the hit rate and false alarm data (see Fig. 3c). We corrected for extreme proportions (i.e., 1 and 0) by using the log-linear rule, whereby a constant of 0.5 was added [3].

**Table 1:** Means and standard deviations for Experiments 1 and 2 across probes and shift conditions

Experiment	Measurement	Associated probe (hit rate)		Dissociated probe (hit rate)		Negative probe (false alarm)	
		Shift	No Shift	Shift	No Shift	Shift	No Shift
1	Hit rate/false alarm	95.50%	94.79%	93.67%	94.72%	8.86%	7.77%
		(6.31%)	(7.39%)	(9.06%)	(5.59%)	(12.91%)	(10.17%)
	Response time (s)	1.674	1.705	1.774	1.852	2.386	2.278
		(0.493)	(0.409)	(0.561)	(0.543)	(0.678)	(0.58)
	*d’*	2.982	3.057	2.914	2.978	N/A	N/A
		(0.83)	(0.781)	(0.867)	(0.784)		
	*C*	− 0.091	− 0.072	− 0.057	− 0.033	N/A	N/A
		(0.243)	(0.292)	(0.294)	(0.216)		
2	Hit rate/false alarm	80.71%	82.29%	82.25%	81.92%	37.93%	30.11%
		(15.43%)	(12.36%)	(15.77%)	(18.99%)	(20.03%)	(19.40%)
	Response Time (s)	3.038	3.106	3.228	3.284	3.552	3.552
		(0.862)	(0.888)	(0.989)	(1.06)	(1.057)	(1.026)
	*d’*	1.261	1.542	1.369	1.596	N/A	N/A
		(0.926)	(0.92)	(1.039)	(1.168)		
	*C*	− 0.284	− 0.19	− 0.338	− 0.217	N/A	N/A
		(0.323)	(0.297)	(0.32)	(0.282)		

We computed a 2 (probe: associated, dissociated) × 2 (shift, no shift) repeated measures ANOVA, with block order as a between-subjects variable of no interest. There were no significant effects of probe (*F*(1,27) = 2.087, *p* = 0.160, η^2^ = 0.065), shift (*F*(1,27) = 0.889, *p* = 0.354, η^2^ = 0.032), nor their interaction (*F*(1,27) = 0.008, *p* = 0.929, η^2^ < 0.001) on d’ (block order was also not significant; *F*(1,27) = 0.974, *p* = 0.333, η^2^ = 0.035). To follow up the null effect of shift, we computed a set of Bayesian paired *t*-tests (using JASP v0.9.2.0, default Cauchy prior width = 0.707) and found that there was sufficient evidence for the null hypothesis for there being no effect of shift on associated (BF_01_ = 3.870) or dissociated (BF_01_ = 3.590) probes. Furthermore, there were no significant effects of probe type (*F*(1,27) = 2.087, *p* = 0.160, η^2^ = 0.065), shift (*F*(1,27) = 0.131, *p* = 0.720, η^2^ = 0.005), or their interaction (*F*(1,27) = 0.008, *p* = 0.929, η^2^ < 0.001) on the *C* bias parameter (that is, the bias towards responding ‘yes’ or ‘no’; see Fig. 3d). Block order was also not significant (*F*(1,27) = 0.175, *p* = 0.679, η^2^ = 0.006). Follow-up Bayesian paired *t*-tests supported the null hypothesis for there being no effect (as opposed to an underpowered effect) of shift on the bias parameter for associated (BF_01_ = 4.693) or dissociated (BF_01_ = 4.710) probes.

The distribution of hit rates across participants and conditions strongly suggested a ceiling effect (mode = 100%). To investigate whether the ceiling effect obscured significant differences between shift and no shift, we analysed just the lowest-performing participants (median split at 95.35% accuracy, producing a subsample with N = 14, M = 88.65%, SD = 6.13%, range = 72.20% to 95.30%). Again, this revealed non-significant effects of shift (*F*(1,12) = 0.196, *p* = 0.666, η^2^ = 0.015), probe (*F*(1,12) = 0.553, *p* = 0.471, η^2^ = 0.041), and their interaction (*F*(1,12) = 1.181, *p* = 0.298, η^2^ = 0.087) on *d’*. Due to the decreased sample size, however, there was only sufficient evidence for there being no effect of shift for associated (BF_01_ = 3.515) but not dissociated (BF_01_ = 1.769) probes.

Finally, we analysed the response time data (see Fig. 3d and Table 1) using the same repeated-measures ANOVA design as for accuracy. Though we did not observe a main effect of shift (*F*(1,27) = 2.934, *p* = 0.098, η^2^ = 0.096), we did find an effect of probe type (*F*(1, 27) = 6.492, *p* = 0.017, η^2^ = 0.193). Response times were faster for associated probes (M = 1.689 s) than dissociated probes (M = 1.813 s; *t*(28) = 2.635, *p* = 0.014). There was no significant interaction between probe type and doorway (*F*(1,27) = 0.100, *p* = 0.754, η^2^ = 0.003). Block order was not significant (*F*(1,27) = 0.909, *p* = 0.349, η^2^ = 0.033).

Overall, these results demonstrate evidence in favour of there being no effect of shift on signal detection sensitivity or response bias for associated or dissociated objects.

### Experiment 2

To address the ceiling effect observed in Experiment 1, we introduced a distractor task that would interfere with object memorisation and thus encourage forgetting. After picking up the object and releasing the trigger (initiating movement), participants had to verbally count backwards, in sixes, from a random number provided by the experimenter until the probe screen appeared. Hence, this task increased working memory load during the period between interacting with the objects on the table to being probed by the question screen.

**Fig. 4:**
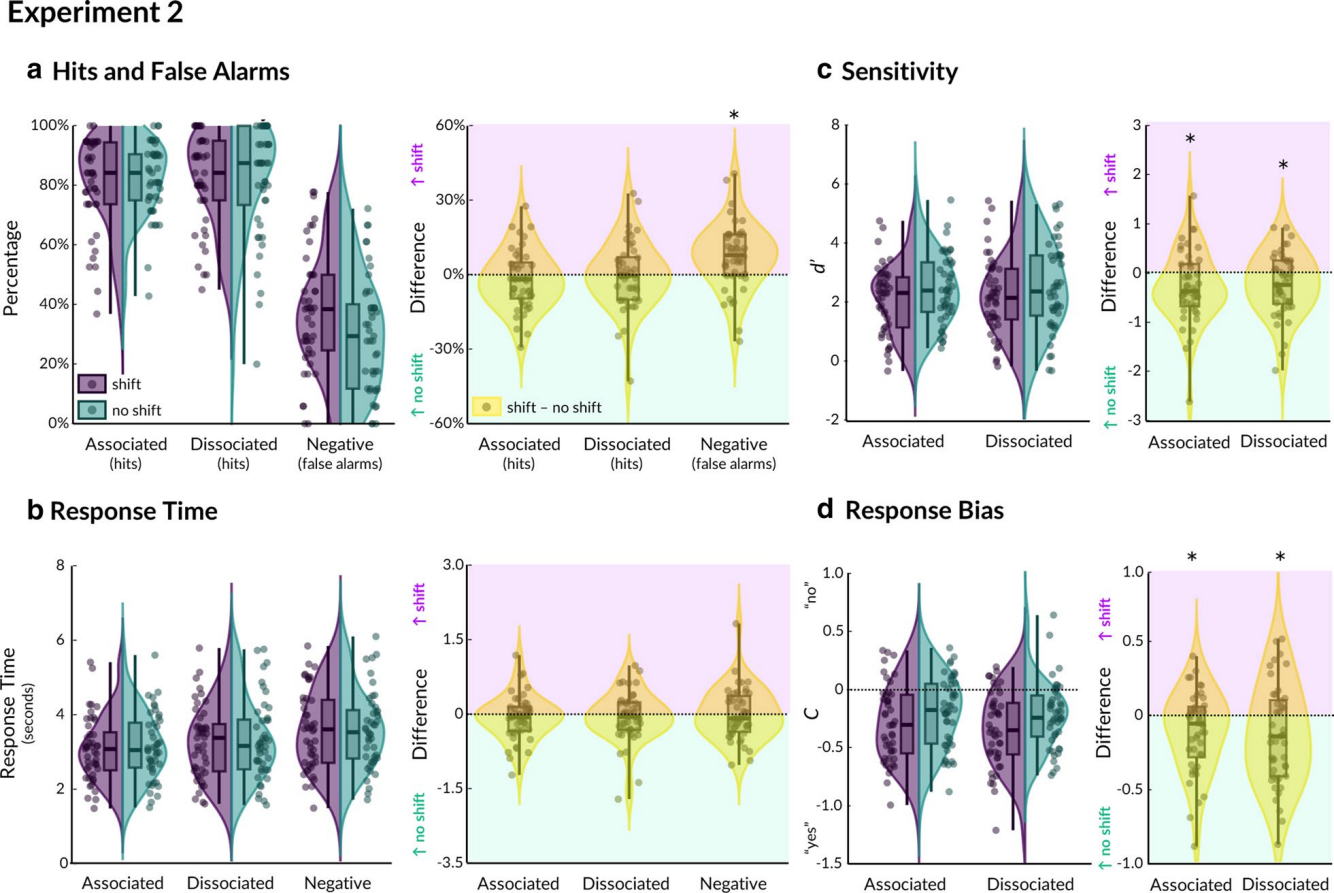
Results from virtual reality Experiment 2. Violin plots are shown for each condition (purple = shift, green = no shift), as well as the difference score per condition (yellow = shift minus no shift). Hits and false alarms are shown in **a**, response times in **b**, signal detection (*d prime*) in **c**, and response bias (*C* parameter) in **d**. Boxplots indicate median, with shaded area for the interquartile range (25th to 75th percentiles), and vertical lines for the minimum and maximum. Dots represent individual participants (*N* = 40). Significant effects of shift (i.e., the “doorway effect”) are indicated by * *p* < .05

As expected, the mean hit rate in Experiment 2 was lower overall at 81.79% (SD = 13.13%, range = 41.71% to 98.68%; see Fig. 4a and Table 1), after removing trials according to the same criteria as Experiment 1 (minimum 13 trials per condition, M = 18, SD = 1.94). The mean false alarm rate across conditions per participant ranged from 0 to 75% (M = 34.02%, SD = 18.50%). We then repeated the same series of analyses as for Experiment 1. First, we computed and analysed *d’* (see Fig. 4c). Unlike in Experiment 1, signal detection was differentially affected for probes following a shift (M = 1.315) than no shift (M = 1.569; F(1,43) = 8.370, p = 0.006, η2 = 0.162). The effect of probe type on *d’* was not significant (*F*(1,38) = 1.885, *p* = 0.177, η_p_^2^ = 0.040), nor was its interaction with shift (*F*(1,43) = 0.318, *p* = 0.576, η^2^ = 0.007). Block order was not significant (*F*(1,43) < 0.001, *p* = 0.979, η^2^ < 0.001). The same analysis on the bias criterion, *C*, revealed that participants were significantly biased towards “yes” responses after a shift (M = -0.311) than after no shift (M = -0.203; *F*(1,43) = 9.364, *p* = 0.004, η^2^ = 0.166; see Fig. 4d; no significant effect of block; *F*(1,43) = 0.150, *p* = 0.700, η^2^ = 0.003). Therefore, going through doorways significantly reduced sensitivity to object probes and induced an overall bias towards reporting “yes”.

To further investigate the nature of this effect, we performed paired t-tests on the hit rates and false alarms separately for associated, dissociated and negative probes, similar to previous studies [15]. Although the accuracy data in Experiment 2 was not as highly negatively skewed (skewness ranged from -1.23 to -0.23) as it was in Experiment 1 due to the reduction of an obvious ceiling effect, the residuals were still not normally distributed (Shapiro–Wilk tests for four out of six conditions were significant: *p* < 0.038). Accordingly, we computed non-parametric two-tailed exact sign tests. This revealed that the doorway effect was significant only for negative (33 participants had lower accuracy after a shift, 9 had higher accuracy, and 3 had no difference, *p* < 0.001) but not associated (28 participants had lower accuracy after a shift, 17 had better accuracy, and 4 showed no difference, *p* = 0.136) or dissociated (19 participants had lower accuracy after a shift, 20 had better accuracy, and 6 showed no difference, *p* = 1) probes. Paired Bayesian t-tests also provided extremely strong evidence for a shift effect on negative probes (BF_10_ = 68.563) and sufficient evidence for the null hypothesis for there being no shift effect on associated (BF_01_ = 4.322) or dissociated (BF_01_ = 6.115) probes. These results indicate that the reduction in *d’* after a shift was predominantly due to there being a higher false alarm rate for negative probes, rather than a reduced hit rate for associated and dissociated probes. Similarly, the significant shift in response criterion towards saying ‘yes’ was primarily due to the increased false alarm rate for negative probes.

Finally, we investigated the response time data and saw an effect of probe type (*F*(1,43) = 7.764, *p* = 0.008, η^2^ = 0.153), such that responses were faster for associated (M = 3.072 s) than dissociated (M = 3.526 s) probes (see Fig. 4b). There was no significant effect of shift (*F*(1,43) = 0.957, *p* = 0.333, η^2^ = 0.022), nor an interaction of shift with probe (*F*(1,43) = 0.070, *p* = 0.793, η^2^ = 0.002). Block order was not significant (*F*(1,43) = 0.001, *p* = 0.971, η^2^ < 0.001).

Overall, the findings from Experiment 2 suggest that, under conditions of working memory load during memorisation time, doorways do impair mnemonic performance but not due to forgetting (i.e., fewer hits and more misses, as typically reported by previous research [15]). Instead, doorways increased the false alarm rate to negative probes.

## Experiments 3 and 4

### Aim

In Experiments 3 and 4, we aimed to conceptually replicate previous experiments that demonstrate the doorway effect in real life contexts [16]. Experiment 3 consisted of passively watching a video from a first-person perspective of someone traversing a corridor with or without curtain boundaries (see Fig. 2). Experiment 4 involved active navigation through the same corridor. The curtain set-up closely resembled that used by a previous study demonstrating the doorway effect during imagined navigation [6]. Also, similar to previous real-life investigations into the doorway effect [6, 10, 12, 16], we increased working memory load demand (counting task) and had participants memorise multiple items to increase task difficulty. We hypothesised that, should the doorway effect be robust to even impermanent boundaries (e.g., curtains) and returning to the original context (as has been shown previously [16]), then participants would demonstrate impaired memory performance after crossing a boundary.

## Methods

### Participants

Experiments 3 and 4 were both conducted at Bond University, Australia. Participants were recruited from the Bond University Research Participation Scheme, as well as from the public via social media. For Experiment 3, a sample of 26 people (12 males, 14 females) aged between 19 and 28 years (M = 23.15, SD = 2.19) participated. For Experiment 4, a (separate) sample of 26 people (2 males, 24 females) aged between 18 and 36 years (M = 21.27, SD = 3.83) participated. The studies in both experiments were approved by the Human Research Ethics Committee at Bond University. All participants gave written consent and received partial course credit for their participation and were also given the chance to win a $50 gift voucher after completing the study.

### Stimuli

Both experiments used a hallway in the behavioural research building at Bond University as the spatial navigation environment. The hallway was 16.45 m long, 2.36 m wide, and 2.35 m high. The hallway contained no furniture, was brightly lit, and had task-irrelevant doors to other rooms on either side (see Fig. 2). To create boundaries for the “shift” condition, blue curtains were hung from the ceiling that segmented the hallway into 3 sections (each section approximately 3.5 m long). The curtains had a split in the middle that hung slightly open.

In Experiment 3, participants viewed a video from a first-person perspective (i.e., filmed from eye-level) that simulated the experience of walking down the hallway and back again, either with curtains for the “shift” condition (two event boundaries, each crossed twice) or without curtains for “no shift” condition. To reduce stimulus repetition and maximise participant engagement, we recorded 5 different videos (approximately 45.2 s duration) of the same walk for each condition (10 videos total) using an iPhone 6 (f/2.2, 8 megapixels), turning either left (2 videos) or right (3 videos) at the end of the hallway.

Participants were required to memorise photographs of butterflies. There were 16 photographs of unique butterfly species. The stimuli were printed in a 4 × 4 grid and subsequently cut out in 10 × 10 cm squares so that they could be arranged in different configurations when presented to the participant. Twenty-five grids were pseudorandomly generated for the experiment.

### Procedure

In Experiment 3, participants were seated in a dark room on a swivel chair at a desk upon which the 16 butterfly stimuli were arranged in a specific grid layout (one of the 25 layouts). After the lights were turned on (revealing the stimuli on the desk), participants were given 30 s to memorise the location of each different butterfly in the grid. After 30 s, the lights were turned off and participants were required to spin their chair around to face an open laptop on a desk behind them. The participant then used the laptop track pad to press play on the video of the hallway walk. Participants were encouraged to imagine they were the person walking down the hallway. Like in Experiment 2, participants were also required to count backwards out loud in decrements of 3  from a random number between 90 and 100 (provided by the experimenter). While the participant watched the video, the experimenter stacked and shuffled the photographs behind the participant. After the video ended, the lights were turned back on. To ensure participants paid attention during the video, the experimenter asked the participant whether they had turned left or right at the end of the hallway (all participants answered 100% correctly). After this, participants were given 45 s to rearrange the butterflies into the grid formation they had memorised. Overall, there were 24 trials that alternated between the shift and no shift condition (the starting condition was counterbalanced across participants).

The procedure for Experiment 4 was essentially the same as Experiment 3, except that the participants actually completed the walk themselves instead of watching a video. Participants memorised the butterfly stimuli for 30 s while seated at a desk at one end of the hallway. The experimenter then collected the stimuli while the participant stood up and completed the walk, counting backwards out loud in decrements of 3  from a random number between 90 and 100 provided by the experimenter. Participants freely chose to turn left or right at the end of the hallway. Upon return, the participants were given 45 s to rearrange the stimuli into the memorised grid formation.

In both Experiments 3 and 4, participants completed an initial practice trial. On each trial, the experimenter recorded the number of stimuli placed by the participant in the correct grid location. Finally, once the experiment was complete, participants were questioned about which condition they believed was more challenging (i.e., which condition they personally believed had made it more difficult for them to remember the stimuli).

## Results

**Fig. 5:**
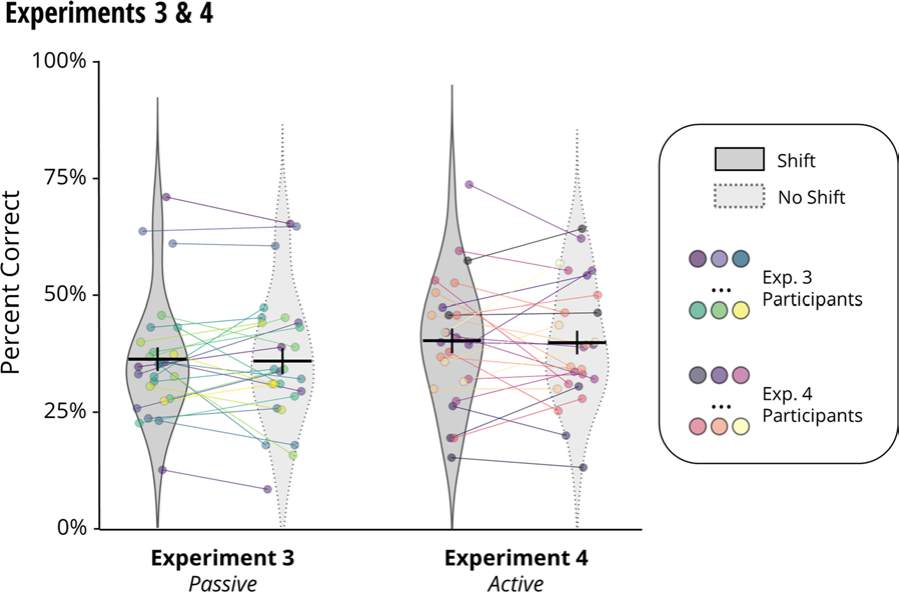
Memory performance in Experiments 3 and 4. Experiment 3 and 4 both made use of a real life hallway environment (with or without a dividing curtain, to establish an event boundary), except that in Experiment 3 participants navigated the hallway passively by viewing a first-person video, and in Experiment 4 participants actively navigated the hallway themselves. Performance on a memory task is illustrated by each graph, depicting shift (dark violin plot with solid boundary, left) and no shift (light violin plot with dotted boundary, right) conditions. The group average is indicated by black horizontal bars (vertical bars represent standard error of the mean). Different participants in each group (Exp. 3 and 4) are represented by coloured points

For both Experiments 3 and 4, we conducted a two-way ANOVA (shift, no shift), with condition order (shift or no shift completed first) as a between-subjects factor, to determine whether memory for the butterfly grid positions had been impaired after passively (Experiment 3) or actively (Experiment 4) experiencing the doorway transitions. Accuracy was calculated as the percentage of correctly placed items out of a possible 16. We found that performance was not significantly different between the shift and no shift conditions in Experiment 3 (M_Δ_ = 0.41%, SD_Δ_ = 7.79%, *F*(1,24) = 0.021, *p* = 0.887, η^2^ = 0.001, BF_10_ = 0.281) or Experiment 4 (M_Δ_ = 0.40%, SD_Δ_ = 9.34%, *F*(1,24) = 0.071, *p* = 0.793, η^2^ = 0.001, BF_10_ = 0.290; see Fig. 5). In Experiment 4, however, there was a significant interaction between shift condition and condition order (*F*(1,24) = 12.864, *p* = 0.001, η^2^ = 0.348), such that accuracy was higher in whichever condition (shift or no shift) participants completed first (shift first: M_S_ = 41.95%, M_NS_ = 36.94%, *t*(12) = -2.705, *p* = 0.019; no shift first: M_S_ = 38.10%, M_NS_ = 43.91%, *t*(12) = 2.439, *p* = 0.031). This suggests either a fatigue effect [1] or proactive interference [5]. There was no significant interaction between shift and condition order in Experiment 3 (*F*(1,24) = 2.762, *p* = 0.110, η^2^ = 0.103, BF_10_ = 0.169). Crucially, condition order was counterbalanced across participants and thus did not confound the shift condition.

Altogether, these results suggest that crossing event boundaries, either imaginatively through watching a video or by actually moving in a real-life environment, did not influence memory, even on a relatively more difficult task (mean accuracy was 36.30% ± 12.85% and 40.22% ± 12.17% for Experiments 3 and 4, respectively).

## Discussion

The doorway effect has been reported by multiple previous studies, each demonstrating a robust medium-to-strong effect across various environmental and cognitive manipulations. The aim of the present study was to investigate the doorway effect under a particular set of constraints. In the first two experiments, a highly immersive and controlled virtual environment was used, with or without working memory load. In the last two experiments, a real-life environment was used, with active (navigation carried out by participant) or passive (navigation was observed from a first-person perspective video) movement.

Contrary to our hypotheses, we observed sufficient evidence for the null hypothesis in all but one of four experiments. In Experiment 2, where the memory task was carried out in a virtual environment with additional working memory load, there was a significant effect of the door on mnemonic performance. Signal detection was reduced and there was a shift in response criterion towards saying “yes”. Further examination, however, showed that doorways did not induce forgetting in the way that has been typically reported by previous studies [15]. In Experiment 2, hit rates were not significantly influenced by doorways, but there were significantly more false alarms to mismatched recognition probes (i.e., negative probes).

The increased false alarm rate suggests that, rather than flushing working memory of the previous event model (resulting in fewer hits), the boundaries, in concert with a secondary counting task, created sufficient cognitive interference in the memory system to effectively reduce the discriminability across encoded objects (resulting in more false alarms). This is in line with some previous studies that also observed more errors to negative probes (e.g., [15, 16]), although many studies have omitted negative probes from the design altogether and thus only report reduced hit rates on shift trials (e.g., [6, 9–11, 17].

The simplicity of the task was reflected by the results of Experiment 1, where accuracy was at ceiling (mean error rate was 6.49%, which translates to approximately 1 mistake per condition). We demonstrated that even the worst-performing participants showed no shift effect, although there was insufficient evidence for dissociated probes. Notably, some previous studies report similarly high accuracies between 80 and 99% [8, 17] and yet still report significant shift effects on hit rates using parametric statistics. This is especially poignant given that the trial numbers in these studies were fewer and unbalanced compared to the current study (e.g., 6 shift trials and 12 no shift trials per probe in [8], compared 20 trials each in the present study). Hence, in previous studies, a 5–10% difference in performance translates to a difference of only 1 to 2 trials. Behavioural patterns generated from few observations are susceptible to spurious artefacts, especially when the same experimental procedure is used for all participants. Hence, our higher-powered counterbalanced design is less likely to be confounded by systematic noise introduced by experimental procedures.

The results from Experiments 1 and 2 suggest that event boundaries interfere with mnemonic performance during passive movement only under conditions of high interference, such as while performing a concurrent counting task. This finding extends knowledge gained from previous studies in multiple ways. Firstly, it suggests that the location updating effect is dependent on working memory load capacity. This relates to the second principle of the event horizon model, which postulates that information from previous event models is less available than information from the current event model because there is a primacy of the current event model in working memory [19]. Under minimal working memory load, there might be a primacy of the current event model but still enough capacity for previous event models, thus resulting in intact memory recognition of the held object within the same or previous room, as seen in Experiment 1. When working memory load is pushed to capacity, however, the previous event model might then become more difficult to access without explicit probing (i.e., the intact colour-shape pair on associated and dissociated probes, as opposed to the mismatched colour-shape pair on negative probes), resulting in impaired signal detection such as that seen in Experiment 2.

The second contribution of our findings is that our highly controlled virtual environment highlights the significance of doorways. Every room in the experiment was essentially identical, meaning that there could be no effects of anticipating a shift during item encoding (i.e., participants could not predict whether they would transition through a door or not while they were seated at the table memorising objects), nor any visual prediction error or attentional capture due to an environmental change [19], such as a new wall pattern [15]. Therefore, Experiment 2 demonstrated that the simple and task-irrelevant visual addition of a doorway significantly increased false alarm rates. Notably, however, there was no significant effect of doorways on hit rates in either of the VR experiments. We speculate that, had the event boundaries been more salient (e.g., changes in room colour), we might have observed the same reduced hit rate after a shift as previous studies [15]. This highlights a potentially fruitful line of investigation into how varying the strength of event boundaries might differentially impact signal and noise distributions, resulting in different impacts on hits and false alarms.

A notable difference between the present VR experiments (1 and 2) and previous studies is that the navigation experience was passive rather than active [8, 9, 12, 15–18]. Previous research, unrelated to the doorway effect, has shown that active navigation enhances memory for the spatial layout, while passive navigation enhances memory for objects [13]. Thus, the effect of doorways on object memory might be reduced in passive navigation paradigms. This also dovetails with the observation by Pettijohn and Radvansky [11] that, during passive virtual navigation, the effect size of the doorway effect was approximately halved. A likely explanation is that active navigation increases engagement and heightens attention to the visuo-spatial environment, which in turn enhances the impact of the location updating effect by strengthening the saliency of event boundaries.

In Experiments 3 and 4, we sought to replicate the doorway effect using a real-life environment, navigated either passively via watching a recorded video (Experiment 3) or actively via actually walking through the environment (Experiment 4). Despite previous research yielding the doorway effect in both forms of interaction [11], we found sufficient evidence in favour of the null hypothesis in both scenarios. This was surprising for a number of reasons. Firstly, we increased task difficulty by imposing greater working memory load (counting task, similar to maths problems given in similar studies; [16]) and increasing the memorised information (16 visually similar items in a specific arrangement). As a result, the mean accuracy (38.26%) was in between chance level (6.25%) and near-perfect performance (one mistake = 87.50%), eliminating floor or ceiling effects. Secondly, previous studies have found that re-crossing multiple boundaries and returning to the original location that items were encoded in impairs memory further [16]. This was not the case in Experiments 3 or 4. Thirdly, interviews with the participants revealed that the majority (approximately 64%) perceived the shift condition as being more difficult than the no shift condition, while only approximately 22% perceived the no shift condition as being more difficult and 14% perceived the conditions as equally difficult.

There are several potential explanations for why the doorway effect did not replicate in Experiments 3 and 4. One is that memory was probed in a different way. In previous studies (including Experiments 1 and 2 here), recognition memory was tested by providing the names of a colour and shape, to which participants responded “yes” or “no”. In Experiments 3 and 4, participants were presented with a shuffled arrangement of the 16 stimuli and required to put them back in the memorised order. Hence, the spatial relations between stimuli were tested, rather than recognition of the stimuli themselves. Such a task is perhaps more similar to familiarity than explicit recognition, the former of which has been shown to be relatively unaffected by the location updating effect [20]. Another potential explanation is that the curtains did not convincingly create event boundaries. Note, however, that this is at odds with other studies using even subtler event boundaries (e.g., transparent doors; [8], imagining navigation along a similar corridor with curtains as boundaries, [6], etc.).

## Conclusions

Overall, our findings across all four experiments suggest that the renowned “doorway effect” is likely to be more nuanced than originally thought, as it only emerged in the form of increased false alarms under considerable working memory load. The same task without working memory load produced no significant effects, nor did a similar memory task implemented in real life with either active or passive interaction. Indeed, this finding resonates more closely with real-life experience, where we might occasionally forget a single item we had in mind after walking into a new room but, crucially, this usually happens when we have other things on our mind, or when we have moved from one distinct context to another.

Our findings reveal that a number of elements are likely crucial for spatial updating to impact recognition memory. In particular, comparing our findings to previous literature reveals that active versus passive navigation, as well as visual context changes, likely augment the doorway effect by increasing the salience of and attention to location changes. Finally, although the focus here was on spatial event boundaries, our findings suggest that other forms of boundaries (e.g., semantic and temporal) are likely to increase false alarm rates to ambiguous recognition probes, while they might more effectively reduce hit rates when the boundaries more clearly delineate between event models (e.g., via increasing the attention to and the salience of the shift).

## Data Availability

The datasets generated and/or analysed during the current study are available in the Open Science Framework repository, https://osf.io/6udbt/.
